# Digital Healthy Diet Literacy and Fear of COVID-19 as Associated with Treatment Adherence and Its Subscales among Hemodialysis Patients: A Multi-Hospital Study

**DOI:** 10.3390/nu15102292

**Published:** 2023-05-12

**Authors:** Lan T. H. Le, Tu T. Tran, Tuyen Van Duong, Loan T. Dang, Trung A. Hoang, Dung H. Nguyen, Minh D. Pham, Binh N. Do, Hoang C. Nguyen, Linh V. Pham, Lien T. H. Nguyen, Hoi T. Nguyen, Nga T. Trieu, Thinh V. Do, Manh V. Trinh, Tung H. Ha, Dung T. Phan, Thao T. P. Nguyen, Kien T. Nguyen, Shwu-Huey Yang

**Affiliations:** 1 Training and Direction of Healthcare Activity Center, Thai Nguyen National Hospital, Thai Nguyen City 241-24, Vietnam; lanhuong.bvtutn@gmail.com; 2 Biochemistry Department, Thai Nguyen National Hospital, Thai Nguyen City 241-24, Vietnam; 3 Director Office, Thai Nguyen National Hospital, Thai Nguyen City 241-24, Vietnam; nguyenconghoang@tnmc.edu.vn; 4 International Ph.D. Program in Medicine, College of Medicine, Taipei Medical University, Taipei 110-31, Taiwan; d142109017@tmu.edu.tw; 5 Department of Internal Medicine, Thai Nguyen University of Medicine and Pharmacy, Thai Nguyen 241-17, Vietnam; 6 School of Nutrition and Health Sciences, Taipei Medical University, Taipei 110-31, Taiwan; sherry@tmu.edu.tw; 7 Faculty of Nursing and Midwifery, Hanoi Medical University, Hanoi 115-20, Vietnam; dangthiloan@hmu.edu.vn; 8 School of Nursing, National Taipei University of Nursing and Health Sciences, Taipei 112-19, Taiwan; 9 Hemodialysis Department, Nephro-Urology-Dialysis Center, Bach Mai Hospital, Hanoi 115-19, Vietnam; hoangtrung.doctor@gmail.com (T.A.H.); nhdungbm@gmail.com (D.H.N.); 10 Department of Nutrition, Military Hospital 103, Hanoi 121-08, Vietnam; ducminh.pham@vmmu.edu.vn; 11 Department of Nutrition, Vietnam Military Medical University, Hanoi 121-08, Vietnam; 12 Department of Military Science, Vietnam Military Medical University, Hanoi 121-08, Vietnam; nhubinh.do@vmmu.edu.vn; 13 Department of Infectious Diseases, Vietnam Military Medical University, Hanoi 121-08, Vietnam; 14 President Office, Thai Nguyen University of Medicine and Pharmacy, Thai Nguyen City 241-17, Vietnam; 15 Department of Pulmonary & Cardiovascular Diseases, Hai Phong University of Medicine and Pharmacy Hospital, Hai Phong 042-12, Vietnam; pvlinh@hpmu.edu.vn (L.V.P.); nthlien@hpmu.edu.vn (L.T.H.N.); 16 President Office, Hai Phong University of Medicine and Pharmacy, Hai Phong 042-12, Vietnam; 17 Director Office, Hai Phong International Hospital, Hai Phong 047-08, Vietnam; hoinguyenthanhbm@gmail.com; 18 Hemodialysis Division, Hai Phong International Hospital, Hai Phong 047-08, Vietnam; bstrieunga@gmail.com; 19 Director Office, Bai Chay Hospital, Ha Long 011-21, Vietnam; dovanthinhhscc@gmail.com; 20 Director Office, Quang Ninh General Hospital, Ha Long 011-08, Vietnam; trinhmanhqnsyt@gmail.com; 21 Director Office, General Hospital of Agricultural, Hanoi 125-16, Vietnam; hahuutung.200564@gmail.com; 22 Faculty of Nursing, Hanoi University of Business and Technology, Hanoi 116-22, Vietnam; phanthidzungvd@gmail.com; 23 Nursing Office, Thien An Obstetrics and Gynecology Hospital, Hanoi 112-06, Vietnam; 24 Institute for Community Health Research, University of Medicine and Pharmacy, Hue University, Hue 491-20, Vietnam; nguyenthiphuongthao@hueuni.edu.vn; 25 Department of Health Promotion, Faculty of Social and Behavioral Sciences, Hanoi University of Public Health, Hanoi 119-10, Vietnam; ntk1@huph.edu.vn; 26 Nutrition Research Center, Taipei Medical University Hospital, Taipei 110-31, Taiwan; 27 Research Center of Geriatric Nutrition, Taipei Medical University, Taipei 110-31, Taiwan

**Keywords:** hemodialysis, fear, COVID-19, physical activity, digital healthy diet literacy, health literacy, treatment adherence, medication, fluid, diet

## Abstract

Treatment adherence (TA) is a critical issue and is under-investigated in hemodialysis patients. A multi-center study was conducted from July 2020 to March 2021 on 972 hemodialysis patients in eight hospitals in Vietnam to explore the factors associated with TA during the COVID-19 pandemic. Data were collected, including socio-demographics, an End-Stage Renal Disease Adherence Questionnaire (ESRD-AQ), 12-item short-form health literacy questionnaire (HLS-SF12), 4-item digital healthy diet literacy scale (DDL), 10-item hemodialysis dietary knowledge scale (HDK), 7-item fear of COVID-19 scale (FCoV-19S), and suspected COVID-19 symptoms (S-COVID19-S). Bivariate and multivariate linear regression models were used to explore the associations. Higher DDL scores were associated with higher TA scores (regression coefficient, B, 1.35; 95% confidence interval, 95%CI, 0.59, 2.12; *p* = 0.001). Higher FCoV-19S scores were associated with lower TA scores (B, −1.78; 95%CI, −3.33, −0.24; *p* = 0.023). In addition, patients aged 60–85 (B, 24.85; 95%CI, 6.61, 43.11; *p* = 0.008) with “very or fairly easy” medication payment ability (B, 27.92; 95%CI, 5.89, 44.95; *p* = 0.013) had higher TA scores. Patients who underwent hemodialysis for ≥5 years had a lower TA score than those who received <5 years of hemodialysis (B, −52.87; 95%CI, −70.46, −35.28; *p* < 0.001). These findings suggested that DDL and FCoV-19S, among other factors, should be considered in future interventions to improve TA in hemodialysis patients.

## 1. Introduction

Globally, approximately 3.8 million people with end-stage renal disease (ESRD) currently rely on renal replacement therapies [1]. Hemodialysis is the most common treatment option worldwide. Renal transplantation and peritoneal dialysis are also applied [2,3]. In Vietnam, there are around 30,000 ESRD patients who are receiving hemodialysis in 2020 [4].

According to World Health Organization, treatment adherence (TA) refers to how well an individual complies with their healthcare provider’s recommendations, which may include taking prescribed medication, following a specific diet, or making lifestyle changes [5]. In hemodialysis patients, non-adherence links to increased morbidity, mortality, healthcare costs, and burden on the healthcare system [6,7,8,9,10,11,12]. Hemodialysis patients must maintain safe potassium and phosphate serum levels to avoid fatal arrhythmia and osteodystrophy [13]. To prevent edema and cardiovascular complications, they must limit fluid intake [14]. Therefore, patients and healthcare providers must strictly follow the treatment protocol/guideline.

The COVID-19 pandemic affects the adherence of ESRD patients to hemodialysis and medication regimens, resulting in a marked increase in the non-adherence rate from 11.7% to 19.5% [15]. A higher fear of COVID-19 score predicts a higher rate of non-adherence to treatment [15]. Increased levels of physical activity are correlated with health-related quality of life in hemodialysis patients (HRQOL) [16], and non-compliance to drug therapy may be associated with worse HRQOL [17]. Hemodialysis patients with sufficient health literacy (HL) had better fluid management and psychological health [18,19,20,21], following treatment recommendations [22]. HL is described as the patient’s capacity to receive, process, communicate, and comprehend fundamental health information and services necessary for making informed health decisions [23,24]. Thus, when patients are fully informed and comprehend what is expected of them, they are better equipped to participate in health-related decisions and are more likely to adhere to regimens that they had a hand in selecting [25,26,27].

Amidst the pandemic, all hemodialysis centers applied strict measures to restrain the spread of COVID-19, such as using masks and restricting family members from visiting patients. The health-related information is provided on digital platforms that require people to access via the internet using digital devices. Thus, HL has become a crucial skill for people to access the application information to manage their health. Digital healthy diet literacy (DDL) has been promoted to encourage individuals to adopt healthier eating behavior, which can strengthen their immune systems. The DDL is an extended concept of HL, as it includes the ability to obtain, understand, assess, and apply digital information related to healthy diets. This ability can lead to better-eating behavior and health outcomes that are essential for containing the pandemic [19,28,29].

Previous studies have investigated TA among hemodialysis patients [10,22,30,31,32,33,34,35], and others focused on HL and fear of COVID-19 [22]. However, a lack of studies mentioned the role of DDL on TA [35]. Therefore, we conducted a multicenter study to investigate the associated factors of TA in hemodialysis patients, where the impacts of DDL and fear were emphasized. We hypothesized that Social -demographic, Fear of COVID-19, physical activity, HL, suspected COVID-19 symptoms (S-COVID19-S), and DDL were associated with TA in hemodialysis patients.

## 2. Materials and Methods

### 2.1. Study Design and Sample

**Figure 1:**
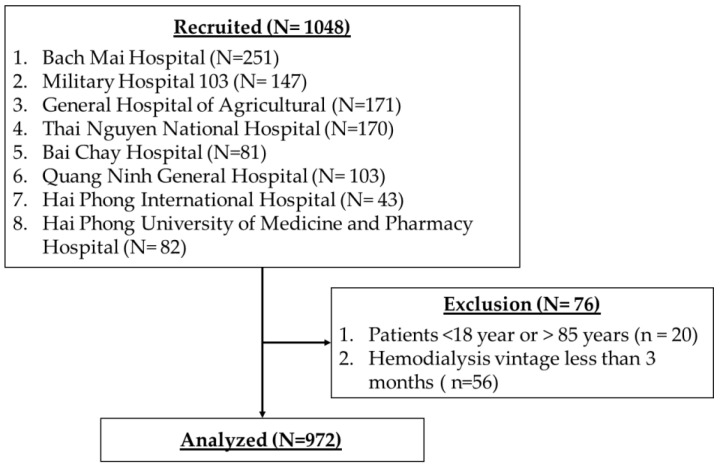
The study sample.

We conducted a cross-sectional study between July 2020 to March 2021 at eight hospitals in Vietnam. Hemodialysis patients were recruited if they were able to read and respond to the survey, aged between 18 and 85 inclusively. Patients are excluded if they received hemodialysis treatment in less than 3 months. During the study period, there was no positive case of COVID-19 among the patients studied. In addition, patients needed to sign the informed consent before participating in the research. The study sample is presented in Figure 1.

### 2.2. Measurements

#### 2.2.1. Treatment Adherence

Treatment adherence (TA) was assessed using the End-Stage Renal Disease Adherence Questionnaire (ESRD-AQ)—46 questions/items—four dimensions (hemodialysis attendance, medication use, fluid restrictions, and diet recommendations). The adherence behavior was calculated using questions numbers 14, 17, 18, 26, 31, and 46, which were further divided into three subscales: adherence to hemodialysis therapy (items 14, 17, and 18), medication (item 26), and fluid and dietary restrictions (items 31 and 46). The responses ranged from 0 to 200. The overall score ranges from 0 to 1200, with higher scores indicating better TA [15,36,37].

#### 2.2.2. Socio-Demographics

Patient’s socio-demographic data (age, gender, education, working status, married status, social status) and medication payment ability were collected. The social status was assessed using a self-reported question “If you self-assess your social status (related to education, occupation, income), which level you are?”. The response options were “low”, middle”, and “high”.

#### 2.2.3. Clinical Parameters

The clinical parameters were assessed, including comorbidity, suspected COVID-19 symptoms (S-COVID-19-S), body mass index (BMI, kg/m^2^), hemodialysis vintage (year), and fear of COVID-19. HD vintage is the length of time on dialysis [38]. The participants were evaluated and classified as having S-COVID-19-S if they presented any of the following symptoms: fever, cough, fatigue, dyspnea, myalgia, sputum production/expectoration, sore throat, runny nose, confusion, headache, chest pain, rhinorrhea, diarrhea, and/or nausea/vomiting [39]. Comorbidities were evaluated using the Charlson comorbidity index (CCI) items [40].

The fear of COVID-19 was measured using the 7-item fear of COVID-19 scale (FCoV-19S) [41]. The responses ranged from 1 to 5 points, with 1 = “strongly disagree” 2 = “disagree”, 3 = “neither agree nor disagree”, 4 = “agree”, and 5 = “strongly agree”. The overall score ranges from 7 to 35, with higher scores indicating more fear.

#### 2.2.4. Health Literacy, Digital Healthy Diet Literacy, and Hemodialysis Diet Knowledge

Participants’ HL and DDL were evaluated using the 12-item short form of the health literacy questionnaire (HLS-SF12) [42] and the digital healthy diet literacy (DDL-4) [19], respectively. The responses range from 1 to 4, with 1 = “very difficult”, 2 = “fairly difficult”, 3 = “fairly easy”, and 4 = “very easy”. These scales have been validated and are commonly applied in Vietnam [21,43,44,45]. The higher index scores indicate better HL or DDL levels [19,46].

The hemodialysis dietary knowledge (HDK) scale was used to examine the participants’ knowledge about the hemodialysis diet. This scale consists of 10 questions on water, potassium, phosphorus, sodium, and protein [47]. Each question consists of three options, including “correct”, “incorrect”, and “not sure”. The correct answer was treated as “correct”, and incorrect or “not sure” answers were treated as “incorrect”. The responses range from 0 to 1, with 0 = “incorrect answer” and 1 = “correct answer”. The HDK total score ranges from 0 to 10, with higher scores indicating greater knowledge. This questionnaire has been validated and utilized in prior research [21,45].

### 2.3. Data Collection Procedure

The procedure was mentioned in previous research [21,45]. Nephrologists, nurses, and students were trained in data collection. The COVID-19 controlling measures were implemented, including mask wearing, hand washing, and physical distancing. About 30 min were spent conducting the survey. The data were then coded, cleaned, and analyzed.

### 2.4. Data Analysis

First, the distributions of the studied variables were checked and presented with a number (n), percentage (%), mean, and standard deviation (SD) appropriately. Next, the *t*-test or one-way ANOVA test was used to explore group differences in treatment adherence scores. The assumption about the distribution of TA is presented in Supplementary Figure S1. Thirdly, we used bivariate linear regression analysis to identify the associated factor of TA. There are several factors that associate with TA. To minimize the residual effects of studied factors, we included those factors based on biological plausibility and prior literature [48,49] into the models. According to the simulation study of confounder-selection strategies, to eliminate the residual effects of studied factors, the factors associated with TA at *p*-value < 0.2 in the bivariate model were included in the multivariate model [50]. The independent variables’ correlations were checked before running the multivariate linear regression to avoid multicollinearity. The Spearman correlation coefficients of independent variables that were less than 0.3 were accepted to be added to the multivariate model (Supplementary Tables S1–S5). All statistical analyses were performed using IBM SPSS Version 26.0 (IBM Corp., Armonk, NY, USA). Statistical significance was established at *p* < 0.05.

## 3. Results

### 3.1. Participants’ Socio-Demographics

The participants’ characteristics are described in Table 1. Among 972 patients, 517 (53.46%) were male, and 450 (46.54%) were female. The number of patients aged 18 to 59 is 585 (60.19%). The treatment adherence was significantly different in some variables, such as education, social status, medication payment ability, suspected COVID-19 symptoms, and hemodialysis vintage (*p* < 0.05).

### 3.2. Associated Factors of Treatment Adherence

Table 2 shows the results of bivariate and multivariate linear regression models. In the bivariate model, patients had a higher TA score were those with higher education (B, 42.53; 95%CI, 18.87, 66.19; *p* < 0.001), middle and high social status (B, 36.11; 95%CI, 16.38, 55.84; *p* < 0.001), “very or fairly easy” medication payment ability (B, 55.21; 95%CI, 34.50, 75.92; *p* < 0.001), with suspected COVID-19 symptoms (B, 25.46; 95%CI, 0.22, 50.70; *p* = 0.048), with a higher HL score (B, 1.98; 95%CI, 1.03, 2.93; *p* < 0.001), and a higher DDL score (B, 1.58; 95%CI, 0.82, 2.53; *p* < 0.001). Inversely, patients had a lower TA score were those with a longer hemodialysis vintage (>5 years) (B, −59.07; 95%CI, −76.92, −41.23; *p* < 0.001), higher scores of fear of COVID-19 (B, −3.19; 95%CI, −4.68, −1.70; *p* < 0.001), and CCI (B, −6.05; 95%CI, −11.34, −0.75; *p* = 0.025), respectively.

To avoid multicollinearity, the correlations among the variables (*p* < 0.20) were examined (Supplementary Tables S1–S5). The variables selected in the multivariable linear regression models were age, working status, social status, medication payment ability, S-COVID-19-S, hemodialysis vintage, DDL index, fear of COVID-19, and CCI. The results of multivariate analysis show that patients who had a higher TA score were those with older age (from 60 to 85) (B, 24.85; 95%CI, 6.61, 43.11; *p* = 0.008), with “very or fairly easy” medication payment ability (B, 27.92; 95%CI, 5.89, 49.95; *p* = 0.013), a higher DDL score (B, 1.35; 95%CI, 0.59, 2.12; *p* = 0.001), respectively. Inversely, patients who had a lower TA score were those with a longer hemodialysis vintage (>5 years) (B, −52.87; 95%CI, −70.46, −35.28; *p* < 0.001), with more fear of COVID-19 (B, −1.78; 95%CI, −3.33, −0.24; *p* = 0.023), respectively.

In Table 3, patients with a longer hemodialysis vintage were less likely to adhere to hemodialysis therapy (B, −22.73; 95%CI, −33.46, −12.01; *p* < 0.001). Patients with older age (60 to 85) (B, 4.39; 95%CI, 0.63, 8.15; *p* = 0.022), a higher DDL (B, 0.17; 95%CI, 0.01, 0.33; *p* = 0.036) were more likely adhere to medication. Inversely, people with S-COVID-19 S (B, −11.06; 95%CI, 15.98, −6.13; *p* = 0.001) and a longer hemodialysis vintage (B, −9.40; 95%CI, −13.01, −5.79; *p* = 0.001) were less likely adhere to medication. In addition, patients who had a higher score of adherence to fluid and diet were those with older age (B, 17.02; 95%CI, 5.04, 29.01; *p* = 0.005), middle and high social status (B, 13.87; 95%CI, 0.25, 27.49; *p* = 0.046), “very and fairly easy” medication payment ability (B, 18.96; 95%CI, 4.12, 33.80; *p* = 0.012), and having suspected COVID-19 symptoms (B, 27.13; 95%CI, 10.78, 43.49; *p* = 0.001), a higher DDL score (B, 1.05, 95%CI, 0.53, 1.56; *p* = 0.001), respectively. Inversely, patients who had a lower score of adherence to fluid and diet were those with a longer hemodialysis vintage (B, −18.70; 95%CI, −30.53, −6.87; *p* = 0.002), fear of COVID-19 (B; −1.70; 95%CI, −2.71, −0.68; *p* = 0.001), and a higher HDK index (B, −4.92; 95%CI, −7.51, −2.34; *p* = 0.001), respectively.

## 4. Discussion

In this study, age, medication payment ability, and DDL were protective factors for treatment adherence in hemodialysis patients during the COVID-19 pandemic. At the same time, fear of COVID-19 and length of hemodialysis (for more than five years) were associated with lower scores of treatment adherence. Therefore, it is vital to design timely education programs for hemodialysis patients to improve health literacy and diet literacy and to allay the fears associated with COVID-19.

The most significant finding in this study is that DDL and HL were protective factors of treatment adherence, especially since this is the first study that shows the effectiveness of DDL in the treatment adherence of HD patients. Health literacy was also associated with better adherence to infection prevention and control during COVID-19 [51]. Addititionally, higher HL and DDL were also positively associated with a healthy diet, physical, and reduced risk of osteoporosis in hemodialysis patients [45,51]. Since DDL is highly correlated with HL, thus DDL as representative of HL was selected into the multivariate model to emphasize its impact. Therefore, the impact of HL cannot be ignored in improving treatment adherence.

We found the role of DDL on adherence to medication, fluid, and diet in particular. During the COVID-19 pandemic, patients relied more on online sources for information; their DDL became a critical factor. Beyond the pandemic, as long as healthy diet information is provided on the online platform, the DDL still keeps a substantial role in promoting healthy eating behavior and health.

In a previous study, HDK was found to have a good association with HL and DDL in terms of mitigating the detrimental effects of S-COVID-19-S on anxious and depressive symptoms in hemodialysis patients [21]. However, HDK has a negative impact on fluid and diet adherence. This could be explained by the fact that the HDK scale tends to represent for diet knowledge of patients [21]; this implies that patients require knowledge and decision-making skills. Therefore, we need to improve the ability of hemodialysis patients to get diet knowledge using digital devices.

Our study shows that fear of COVID-19 is associated with lower scores of treatment adherence. This finding is in line with a prior study, which found that insufficient knowledge and low perception scores, as well as a high fear of COVID-19, were the primary predictors of non-adherence [15]. Furthermore, the fear of COVID-19 also increased the chemotherapy postponement rate in cancer patients [52]. Hemodialysis patients are considered a vulnerable population as they are at an increased risk of COVID-19 infection and experiencing severe outcomes due to several factors such as age, comorbidities (especially diabetes and hypertension), and weakened immune systems due to their underlying health condition [53,54]. Despite orders to remain at home, hemodialysis patients have to leave their homes many times per week for hemodialysis treatment in the dialysis center [55,56]. Taken together, these factors aggravate the non-adherence to treatment in hemodialysis patients.

We analyzed subscales of treatment adherence and found that fear of COVID-19 was associated with adherence to fluids and diet. A previous study showed that hemodialysis patients perceived food and fruit restriction as a complex and challenging process requiring constant effort. The patient’s perception of personal, societal, and systemic barriers made diet and fluid restriction compliance even more difficult. In addition, most patients needed to be adequately supported in managing diet and fluid restriction, and they had established their strategies [57]. Consequently, due to the fear of COVID-19, the non-adherence to the fluid and diet was highly reported.

In our study, S-COVID-19-S was associated with lower scores of medication adherence but associated with higher scores of fluid and diet adherence. When hemodialysis patients have symptoms such as COVID-19, they may need to use other drugs that they may confuse with current medications. Therefore, in practice, health providers should remind patients to pay more attention to regular ESRD-treated medications in addition to the limited-access drugs they obtain when they get symptoms such as worsen COVID-19. Meanwhile, fluid and diet appear to be easier to obtain than medications. Therefore, the patients are likely to adhere to fluid and diet when they have suspected COVID-19 symptoms.

We found that patients with longer hemodialysis vintage had lower scores of treatment adherence. Several studies found that a longer hemodialysis vintage was associated with a lower chance of non-adherence to treatment as a result of individuals examining the impact of dialysis on their bodies and learning to cope with difficulties by engaging with other patients and healthcare providers [58,59]. However, patients with prolonged hemodialysis are prone to polypharmacy, a reflection of the high frequency of comorbidities and the numerous complications associated with dialysis treatment. In addition, previous research suggests that a high number of medications administered to hemodialysis patients is associated with a decline in their quality of life, lower treatment adherence, and an increased mortality risk [60,61]. Particular hemodialysis vintage also was a negative factor in all subscales of treatment adherence. Our study also found that patients with comorbid conditions had less treatment adherence. Together, these rationales could prove our results about the relationship between treatment adherence and hemodialysis duration.

Additionally, patients with better medication payment ability had higher treatment adherence, particularly with fluid and diet adherence. Hemodialysis patients in Vietnam need to co-pay with national health insurance due to the low reimbursement rate of only 25 USD for each session [4]. Moreover, patients frequently face decreased productivity due to the rigorous treatment schedule and their physical limitations. As a result, the worldwide employment rate and income have declined [62,63]. Financial difficulty affects their daily life and physical and mental health, demonstrating a link between financial hardship and overall symptom burden, including depression, fatigue, pain, and sexual dysfunction [64].

We found that older patients adhere to medication, fluid, and diet better than younger patients. This result is similar to previous results [65]. Patients have been living with ESRD for a longer time and have more experience in adhering to the hemodialysis diet and treatment. In addition, older individuals tend to consume low-sodium diets [66].

There are some limitations to consider. First, a cross-sectional study is not possible to establish a causal relationship between studied variables. However, the results of this study may still be useful in developing public health interventions to improve treatment adherence for individuals who are particularly fearful of COVID-19 during the pandemic. Secondly, our data were collected through the survey; thus, it is hard to avoid recall bias. However, our collectors were well-trained and used face-to-face interviews that can reduce recall bias. Thirdly, our study focuses on treatment adherence behaviors through six questions from the ESRD-AQ. Therefore, in the future, we intend to evaluate comprehensive treatment adherence in hemodialysis patients.

## 5. Conclusions

Digital health diet literacy (DDL) and fear of COVID-19 (FCoV-19S) are important factors in determining treatment adherence among hemodialysis patients. Therefore, it is recommended that future interventions to improve treatment adherence among hemodialysis patients should take into account these two factors, particularly during the current and future pandemics.

## Figures and Tables

**Table 1:** Patients’ characteristics and treatment adherence (N = 972).

Variables	Total N (%)	Treatment Adherence (Mean ± SD)	*p*
Age			0.050 ^a^
18–59	585 (60.19)	1040.50 ± 143.47	
60–85	387 (39.81)	1058.93 ± 142.92	
Gender			0.544 ^a^
Male	517 (53.46)	1050.15 ± 143.51	
Female	450 (46.54)	1044.54 ± 143.63	
Education			<0.001 ^b^
Illiterate or elementary	372 (42.32)	1047.86 ±137.61	
Junior high school	281 (31.97)	1018.54 ± 152.53	
Senior high school or above	226 (25.71)	1090.40 ± 139.12	
Working status			0.199 ^a^
Not working	334 (34.97)	1039.38 ± 136.62	
Working	621 (65.03)	1051.84 ± 145.94	
Married status			0.639 ^a^
Never married	86 (8.95)	1040.47 ± 145.99	
Ever married	875 (91.05)	1048.11 ± 143.55	
Social status			<0.001 ^a^
Low	274 (28.81)	1022.39 ± 141.05	
Middle and high	677 (71.19)	1058.51 ± 140.14	
Medication payment ability			<0.001 ^a^
Very or fairly difficult	726 (75.86)	1035.26 ± 143.07	
Very or fairly easy	231(24.14)	1090.47 ± 128.39	
S-COVID-19-S			0.048 ^a^
Without S-COVID-19-S	146 (15.02)	1026.19 ± 142.31	
With S-COVID-19-S	826 (84.98)	1051.66 ± 143.41	
BMI, kg/m^2^			0.468 ^a^
BMI < 24	827 (90.19)	1045.82 ± 140.53	
BMI ≥ 24	90 (9.81)	1057.28 ± 156.74	
HD vintage, year			<0.001 ^a^
<5	550 (56.58)	1073.48 ± 136.81	
≥5	422 (43.42)	1017.41 ± 145.19	
CCI (Mean ± SD)	1.74 ± 1.70		
HL index (Mean ± SD)	23.77 ± 9.44		
DDL index (Mean ± SD)	23.52 ± 11.68		
HDK (Mean ± SD)	4.29 ± 2.30		
FCoV-19S (Mean ± SD)	20.58 ± 6.0		
Treatment adherence (Mean ± SD)	1047.83 ± 143.46		
Hemodialysis treatment (Mean ± SD)	563.32 ± 77.05		
Medication (Mean ± SD)	184.19 ± 27.44		
Fluid and Diet (Mean ± SD)	301.08 ± 97.0		

Abbreviation: SD, standard deviation; S-COVID-19-S, suspected COVID-19 symptoms; BMI, body mass index; HD, hemodialysis; CCI, Charlson comorbidity index; HL, health Literacy; DDL, digital health diet literacy; HDK, hemodialysis dietary knowledge; FCoV-19S, fear of COVID-19 scale. ^a^ Results of the chi-square test; ^b^ Results of one-way ANOVA.

**Table 2:** Associated factors of treatment adherence via bivariate and multivariate linear regression analysis.

Variables	Treatment Adherence			
	Bivariate		Multivariate	
	B (95% CI)	*p*	B (95% CI)	*p*
Age				
18–59				
60–85	18.42 (0.01, 36.85)	0.05	24.85 (6.61, 43.11)	0.008 ^a^
Gender				
Male				
Female	−5.61 (−23.78, 12.54)	0.544		
Education				
Illiterate or elementary				
Junior high school	−29.32 (−51.48, −7.15)	0.01		
Senior high school or above	42.53 (18.87, 66.19)	<0.001		
Working status				
Not working				
Working	12.45 (−6.55, 31.46)	0.199	−2.25 (−21.15, 16.65)	0.815 ^a^
Married status				
Never married				
Ever married	7.63 (−24.25, 39.51)	0.639		
Social status				
Low				
Middle and high	36.11 (16.38, 55.84)	<0.001	19.22 (−1.23, 39.69)	0.066 ^a^
Medication payment ability				
Very or fairly difficult				
Very or fairly easy	55.21 (34.50, 75.92)	<0.001	27.92 (5.89, 49.95)	0.013 ^a^
S-COVID-19-S				
Without S-COVID-19-S				
With S-COVID-19-S	25.46 (0.22, 50.70)	0.048	15.58 (−9.41, 40.58)	0.221 ^a^
BMI, kg/m^2^				
BMI < 24				
BMI ≥ 24	11.46 (−19.50, 42.44)	0.468		
HD vintage, year				
<5				
≥5	−59.07 (−76.92, −41.23)	<0.001	−52.87 (−70.46, −35.28)	<0.001 ^a^
CCI	−6.05 (−11.34, −0.75)	0.025	−3.70 (−9.11, 1.70)	0.179 ^a^
HL index	1.98 (1.03, 2.93)	<0.001		
DDL index	1.58 (0.82, 2.53)	<0.001	1.35 (0.59, 2.12)	0.001 ^a^
HDK index	0.04 (−3.89, 3.96)	0.985		
FCoV-19S	−3.19 (−4.68, −1.70)	<0.001	−1.78 (−3.33, −0.24)	0.023 ^a^

Abbreviations: B, regression coefficient; CI, confidence interval; S-COVID-19-S, suspected COVID-19 symptoms; BMI, body mass index; HD, Hemodialysis; CCI, Charlson comorbidity index; HL, health literacy; DDL, digital health diet literacy; HDK, hemodialysis dietary knowledge; FCoV-19S, fear of COVID-19 scale. ^a^ Multilinear regression model consisting of age, working status, social status, medication payment ability, S-COVID-19-S, HD vintage, DDL index, Fear of COVID-19, and CCI.

**Table 3:** Associate factors of adherence to hemodialysis treatment, medication, fluid, and diet via multivariate linear regression analysis.

	Hemodialysis Treatment		Medication		Fluid and Diet	
	B (95% CI)	*p*	B (95% CI)	*p*	B (95% CI)	*p*
Age						
19–59						
60–85			4.39 (0.63, 8.15)	0.022 ^b^	17.02 (5.04, 29.01)	0.005 ^c^
Gender						
Male						
Female	−4.55 (−15.14, 6.03)	0.398 ^a^				
Education						
Illiterate or elementary						
Junior high school						
Senior high school or above	−1.71 (−8.41, 5.01)	0.618 ^a^	1.86 (−0.45, 0.18)	0.115 ^b^		
Working status						
Not working						
Working					1.36 (−11.23, 13.96)	0.832 ^c^
Married status						
Never married						
Ever married						
Social status						
Low						
Middle and high					13.87 (0.25, 27.49)	0.046 ^c^
Medication payment ability						
Very or fairly difficult						
Very or fairly easy	5.86 (−6.64, 18.37)	0.357 ^a^	2.78 (−1.55, 7.12)	0.208 ^b^	18.96 (4.12, 33.80)	0.012 ^c^
S-COVID-19-S						
Without S-COVID-19-S						
With S-COVID-19-S			−11.06 (−15.98, −6.13)	<0.001 ^b^	27.13 (10.78, 43.49)	0.001 ^c^
BMI, kg/m^2^						
BMI < 24						
BMI ≥ 24						
HD vintage, year						
<5						
≥5	−22.73 (−33.46, −12.01)	<0.001 ^a^	−9.40 (−13.01, −5.79)	<0.001 ^b^	−18.70 (−30.53, −6.87)	0.002 ^c^
CCI	−2.46 (−5.49, 0.56)	0.111 ^a^	0.31 (−0.77, 1.38)	0.581 ^b^		
HL index						
DDL index			0.17 (0.01, 0.33)	0.036 ^b^	1.05 (0.53, 1.56)	0.001 ^c^
HDK	1.9 (−0.3, 4.3)	0.092 ^a^	0.57 (−0.20, 1.35)	0.148 ^b^	−4.92 (−7.51, −2.34)	0.001 ^c^
FCoV-19S			−0.18 (−0.49, 0.12)	0.238 ^b^	−1.70 (−2.71, −0.68)	0.001 ^c^

Abbreviations: B, regression coefficient; CI, confidence interval; S-COVID-19-S, suspected COVID-19 symptoms; BMI, body mass index; HD, hemodialysis; CCI, Charlson comorbidity index; HL, health literacy; DDL, digital health diet literacy; HDK, hemodialysis dietary knowledge; FCoV-19S, fear of COVID-19 scale. ^a^ Multiple linear regression model consisting of gender, education, medication payment ability, HD vintage, HDK, and CCI. ^b^ Multiple linear regression model consisting of age, education, medication payment ability, S-COVID-19-S, HD vintage, DDL index, HDK, Fear of COVID-19, and CCI. ^c^ Multiple linear regression model consisting of age, working status, social status, medication payment ability, S-COVID-19-S, HD vintage, DDL index, HDK index, and Fear of COVID-19.

## Data Availability

Data will be available upon reasonable request to the corresponding author.

## Supplementary Materials

The following supporting information can be downloaded at: https://www.mdpi.com/article/10.3390/nu15102292/s1, Figure S1: The assumption checked for linear regression models of treatment adherence; Table S1: The correlations between the independent variable (Treatment Adherence); Table S2: Associate factors of adherence to hemodialysis treatment, medication, fluid, and diet via bivariate linear regression analysis; Table S3: The correlations between the independent variable (Hemodialysis treatment); Table S4: The correlations between the independent variable (Medication treatment); Table S5: The correlations between the independent variable (Fluid and Diet).

## References

[1] Liyanage T., Ninomiya T., Jha V., Neal B., Patrice H.M., Okpechi I., Zhao M.H., Lv J., Garg A.X., Knight J. (2015). Worldwide access to treatment for end-stage kidney disease: A systematic review. Lancet.

[2] Thurlow J.S., Joshi M., Yan G., Norris K.C., Agodoa L.Y., Yuan C.M., Nee R. (2021). Global epidemiology of end-stage kidney disease and disparities in kidney replacement therapy. Am. J. Nephrol..

[3] Bhargava V., Jasuja S., Tang S.C.W., Bhalla A.K., Sagar G., Jha V., Ramachandran R., Sahay M., Alexander S., Vachharajani T. (2021). Peritoneal dialysis: Status report in south and south east Asia. Nephrology.

[4] Pham Van B., Vo Duc C. (2020). Global Dialysis Perspective: Vietnam. Kidney360.

[5] World Health Organization (2003). Adherence to Long-Term Therapies: Evidence for Action. http://apps.who.int/iris/bitstream/handle/10665/42682/9241545992.pdf;jsessionid=69DD547138169C24724B8622BF4276BD?sequence=1..

[6] Kugler C., Maeding I., Russell C.L. (2011). Non-adherence in patients on chronic hemodialysis: An international comparison study. Survival.

[7] Walser M. (2000). Is there a role for protein restriction in the treatment of chronic renal failure?. Blood Purif..

[8] Unruh M.L., Evans I.V., Fink N.E., Powe N.R., Meyer K.B. (2005). Skipped treatments, markers of nutritional nonadherence, and survival among incident hemodialysis patients. Am. J. Kidney Dis..

[9] Tentori F., Hunt W.C., Rohrscheib M., Zhu M., Stidley C.A., Servilla K., Miskulin D., Meyer K.B., Bedrick E.J., Johnson H.K. (2007). Which targets in clinical practice guidelines are associated with improved survival in a large dialysis organization?. J. Am. Soc. Nephrol..

[10] Bame S.I., Petersen N., Wray N.P. (1993). Variation in hemodialysis patient compliance according to demographic characteristics. Soc. Sci. Med..

[11] Shirazian S., Crnosija N., Weinger K., Jacobson A.M., Park J., Tanenbaum M.L., Gonzalez J.S., Mattana J., Hammock A.C. (2016). The self-management experience of patients with type 2 diabetes and chronic kidney disease: A qualitative study. Chronic Illn..

[12] Naderifar M., Tafreshi M.Z., Ilkhani M., Akbarizadeh M.R., Ghaljaei F. (2018). Correlation between quality of life and adherence to treatment in hemodialysis patients. J. Ren. Inj. Prev..

[13] de Rooij E.N.M., Dekker F.W., Le Cessie S., Hoorn E.J., de Fijter J.W., Hoogeveen E.K. (2022). Serum Potassium and Mortality Risk in Hemodialysis Patients: A Cohort Study. Kidney Med..

[14] Canaud B., Chazot C., Koomans J., Collins A. (2019). Fluid and hemodynamic management in hemodialysis patients: Challenges and opportunities. J. Bras. Nefrol..

[15] Sultan B.O., Fouad A.M., Zaki H.M. (2022). Adherence to hemodialysis and medical regimens among patients with end-stage renal disease during COVID-19 pandemic: A cross-sectional study. BMC Nephrol..

[16] Wu Y.H., Hsu Y.J., Tzeng W.C. (2022). Physical Activity and Health-Related Quality of Life of Patients on Hemodialysis with Comorbidities: A Cross-Sectional Study. Int. J. Env. Res. Public Health.

[17] Alves K.B., Guilarducci N.V., Santos T.d.R., Baldoni A.O., Otoni A., Pinto S.W.L., Zanette C., Sanches C. (2018). Is quality of life associated with compliance to pharmacoterapy in patients with chronic kidney disease undergoing maintenance hemodialysis?. Einstein.

[18] Sørensen K., Van den Broucke S., Fullam J., Doyle G., Pelikan J., Slonska Z., Brand H. (2012). Health literacy and public health: A systematic review and integration of definitions and models. BMC Public Health.

[19] Duong T.V., Pham K.M., Do B.N., Kim G.B., Dam H.T., Le V.-T.T., Nguyen T.T., Nguyen H.T., Nguyen T.T., Le T.T. (2020). Digital healthy diet literacy and self-perceived eating behavior change during COVID-19 pandemic among undergraduate nursing and medical students: A rapid online survey. Int. J. Environ. Res. Public Health.

[20] Chen C., Zheng J., Driessnack M., Liu X., Liu J., Liu K., Peng J., You L. (2021). Health literacy as predictors of fluid management in people receiving hemodialysis in China: A structural equation modeling analysis. Patient Educ. Couns..

[21] Dang L.T., Luong T.C., Nguyen D.H., Hoang T.A., Nguyen H.T., Nguyen H.C., Duong T.H., Tran T.T., Pham L.V., Ngo T.V. (2022). The Associations of Suspected COVID-19 Symptoms with Anxiety and Depression as Modified by Hemodialysis Dietary Knowledge: A Multi-Dialysis Center Study. Nutrients.

[22] Indino K., Sharp R., Esterman A. (2019). The effect of health literacy on treatment adherence in maintenance haemodialysis patients: A cross-sectional study. Ren. Soc. Australas. J..

[23] Baker D.W. (2006). The meaning and the measure of health literacy. J. Gen. Intern. Med..

[24] Wolf M.S., Davis T.C., Osborn C.Y., Skripkauskas S., Bennett C.L., Makoul G. (2007). Literacy, self-efficacy, and HIV medication adherence. Patient Educ. Couns..

[25] DiMatteo M.R., Haskard-Zolnierek K.B., Martin L.R. (2012). Improving patient adherence: A three-factor model to guide practice. Health Psychol. Rev..

[26] Makoul G., Clayman M.L. (2006). An integrative model of shared decision making in medical encounters. Patient Educ. Couns..

[27] Miller T.A. (2016). Health literacy and adherence to medical treatment in chronic and acute illness: A meta-analysis. Patient Educ. Couns..

[28] Iddir M., Brito A., Dingeo G., Fernandez Del Campo S.S., Samouda H., La Frano M.R., Bohn T. (2020). Strengthening the immune system and reducing inflammation and oxidative stress through diet and nutrition: Considerations during the COVID-19 crisis. Nutrients.

[29] Calder P.C., Carr A.C., Gombart A.F., Eggersdorfer M. (2020). Optimal nutritional status for a well-functioning immune system is an important factor to protect against viral infections. Nutrients.

[30] Leggat J., Orzol S.M., Hulbert-Shearon T.E., Golper T.A., Jones C.A., Held P.J., Port F.K. (1998). Noncompliance in hemodialysis: Predictors and survival analysis. Am. J. Kidney Dis..

[31] De Brito Ashurst I., Dobbie H. (2003). A randomized controlled trial of an educational intervention to improve phosphate levels in hemodialysis patients. J. Ren. Nutr..

[32] Sharp J., Wild M.R., Gumley A.I., Deighan C.J. (2005). A cognitive behavioral group approach to enhance adherence to hemodialysis fluid restrictions: A randomized controlled trial. Am. J. Kidney Dis..

[33] Denhaerynck K., Manhaeve D., Dobbels F., Garzoni D., Nolte C., De Geest S. (2007). Prevalence and consequences of nonadherence to hemodialysis regimens. Am. J. Crit. Care.

[34] Naalweh K.S., Barakat M.A., Sweileh M.W., Al-Jabi S.W., Sweileh W.M., Zyoud S.e.H. (2017). Treatment adherence and perception in patients on maintenance hemodialysis: A cross–Sectional study from Palestine. BMC Nephrol..

[35] Duong C.M., Olszyna D.P., Nguyen P.D., McLaws M.-L. (2015). Challenges of hemodialysis in Vietnam: Experience from the first standardized district dialysis unit in Ho Chi Minh City. BMC Nephrol..

[36] Kim Y., Evangelista L.S., Phillips L.R., Pavlish C., Kopple J.D. (2010). The End-Stage Renal Disease Adherence Questionnaire (ESRD-AQ): Testing the psychometric properties in patients receiving in-center hemodialysis. Nephrol. Nurs. J..

[37] Poveda V., Amado L., Filgueiras M., Teixeira L., Miranda V., Santos-Silva A., Paúl C., Costa E. (2016). End-stage renal disease adherence questionnaire: Translation and validation to the portuguese language. Ren. Fail..

[38] Chertow G.M., Johansen K.L., Lew N., Lazarus J.M., Lowrie E.G. (2000). Vintage, nutritional status, and survival in hemodialysis patients. Kidney Int..

[39] BMJ Best Practice (2020). Coronavirus Disease 2019 (COVID-19), History and Exam. https://bestpractice.bmj.com/topics/en-gb/3000201/history-exam.

[40] Hemmelgarn B.R., Manns B.J., Quan H., Ghali W.A. (2003). Adapting the Charlson Comorbidity Index for use in patients with ESRD. Am. J. Kidney Dis..

[41] Nguyen H.T., Do B.N., Pham K.M., Kim G.B., Dam H.T., Nguyen T.T., Nguyen T.T., Nguyen Y.H., Sørensen K., Pleasant A. (2020). Fear of COVID-19 scale—Associations of its scores with health literacy and health-related behaviors among medical students. Int. J. Environ. Res. Public Health.

[42] Duong T.V., Aringazina A., Kayupova G., Nurjanah f., Pham T.V., Pham K.M., Truong T.Q., Nguyen K.T., Oo W.M., Su T.T. (2019). Development and validation of a new short-form health literacy instrument (HLS-SF12) for the general public in six Asian countries. HLRP Health Lit. Res. Pract..

[43] Duong T.V., Nguyen T.T., Pham K.M., Nguyen K.T., Giap M.H., Tran T.D., Nguyen C.X., Yang S.-H., Su C.-T. (2019). Validation of the short-form health literacy questionnaire (HLS-SF12) and its determinants among people living in rural areas in Vietnam. Int. J. Environ. Res. Public Health.

[44] Van Hoa H., Giang H.T., Vu P.T., Van Tuyen D., Khue P.M. (2020). Factors associated with health literacy among the elderly people in Vietnam. BioMed Res. Int..

[45] Le L.T.H., Dang L.T., Wang T.J., Do T.G., Nguyen D.H., Hoang T.A., Pham M.D., Do B.N., Nguyen H.C., Tran T.T. (2022). Osteoporosis Risk in Hemodialysis Patients: The Roles of Gender, Comorbidities, Biochemical Parameters, Health and Diet Literacy. Nutrients.

[46] Sørensen K., Pelikan J.M., Röthlin F., Ganahl K., Slonska Z., Doyle G., Fullam J., Kondilis B., Agrafiotis D., Uiters E. (2015). Health literacy in Europe: Comparative results of the European health literacy survey (HLS-EU). Eur. J. Public Health.

[47] Ryu H., Jeon H.J., Sun H.-K., Han K.H., Whang C.G., Han S.Y. (2014). Repeated education improves diet compliance in maintenance Hemodialysis Patients. Int. J. Urol. Nephrol.

[48] Gast A., Mathes T. (2019). Medication adherence influencing factors—An (updated) overview of systematic reviews. Syst. Rev..

[49] Jain D., Green J.A. (2016). Health literacy in kidney disease: Review of the literature and implications for clinical practice. World J. Nephrol..

[50] Maldonado G., Greenland S. (1993). Simulation study of confounder-selection strategies. Am. J. Epidemiol..

[51] Do B.N., Tran T.V., Phan D.T., Nguyen H.C., Nguyen T.T., Nguyen H.C., Ha T.H., Dao H.K., Trinh M.V., Do T.V. (2020). Health literacy, ehealth literacy, adherence to infection prevention and control procedures, lifestyle changes, and suspected COVID-19 symptoms among health care workers during lockdown: Online survey. J. Med. Internet Res..

[52] Karacin C., Bilgetekin I., B Basal F., Oksuzoglu O.B. (2020). How does COVID-19 fear and anxiety affect chemotherapy adherence in patients with cancer. Future Oncol..

[53] Valeri A.M., Robbins-Juarez S.Y., Stevens J.S., Ahn W., Rao M.K., Radhakrishnan J., Gharavi A.G., Mohan S., Husain S.A. (2020). Presentation and Outcomes of Patients with ESKD and COVID-19. J. Am. Soc. Nephrol..

[54] Fisher M., Yunes M., Mokrzycki M.H., Golestaneh L., Alahiri E., Coco M. (2020). Chronic hemodialysis patients hospitalized with COVID-19: Short-term outcomes in the Bronx, New York. Kidney360.

[55] Okoro R.N. (2021). COVID-19 pandemic: The role of community pharmacists in chronic kidney disease management supportive care. Res. Soc. Adm. Pharm..

[56] Weiss S., Bhat P., Del Pilar Fernandez M., Bhat J.G., Coritsidis G.N. (2020). COVID-19 Infection in ESKD: Findings from a Prospective Disease Surveillance Program at Dialysis Facilities in New York City and Long Island. J. Am. Soc. Nephrol..

[57] Özkan İ., Taylan S. (2022). Diet and fluid restriction experiences of patients on hemodialysis: A meta-synthesis study. Rev. Nefrol. Diálisis Transpl..

[58] Chan Y.M., Zalilah M.S., Hii S.Z. (2012). Determinants of compliance behaviours among patients undergoing hemodialysis in Malaysia. PLoS ONE.

[59] Ozen N., Cinar F.I., Askin D., Mut D., Turker T. (2019). Nonadherence in Hemodialysis Patients and Related Factors: A Multicenter Study. J. Nurs. Res..

[60] Tozawa M., Iseki K., Iseki C., Oshiro S., Higashiuesato Y., Yamazato M., Tomiyama N., Tana T., Takishita S. (2002). Analysis of drug prescription in chronic haemodialysis patients. Nephrol. Dial. Transplant..

[61] Chiu Y.-W., Teitelbaum I., Misra M., De Leon E.M., Adzize T., Mehrotra R. (2009). Pill burden, adherence, hyperphosphatemia, and quality of life in maintenance dialysis patients. Clin. J. Am. Soc. Nephrol..

[62] Walker R.C., Howard K., Tong A., Palmer S.C., Marshall M.R., Morton R.L. (2016). The economic considerations of patients and caregivers in choice of dialysis modality. Hemodial. Int..

[63] Hallab A., Wish J.B. (2018). Employment among patients on dialysis: An unfulfilled promise. Clin. J. Am. Soc. Nephrol. CJASN.

[64] Ng M.S.N., Chan D.N.S., Cheng Q., Miaskowski C., So W.K.W. (2021). Association between Financial Hardship and Symptom Burden in Patients Receiving Maintenance Dialysis: A Systematic Review. Int. J. Env. Res. Public Health.

[65] Clark-Cutaia M.N., Ren D., Hoffman L.A., Burke L.E., Sevick M.A. (2014). Adherence to hemodialysis dietary sodium recommendations: Influence of patient characteristics, self-efficacy, and perceived barriers. J. Ren. Nutr..

[66] Foote J.A., Giuliano A.R., Harris R.B. (2000). Older adults need guidance to meet nutritional recommendations. J. Am. Coll. Nutr..

