# Supplementary material for: Digital Healthy Diet Literacy and Fear of COVID-19 as Associated with Treatment Adherence and Its Subscales among Hemodialysis Patients: A Multi-Hospital Study
Source: Nutrients. 2023 May 12;15(10):2292. doi: 10.3390/nu15102292 (PMC10222703; doi:10.3390/nu15102292)
Supplement: Supplementary file 1 [file nutrients-15-02292-s001.zip › nutrients-2362978-supplementary.pdf]

nutrients

Supplementary Materials

Digital Healthy Diet Literacy and Fear of COVID-19 as Associated with Treatment Adherence and Its Subscales among Hemodialysis Patients: A Multi-Hospital Study

Lan T. H. Le, Tu T. Tran, Tuyen Van Duong, Loan T. Dang, Trung A. Hoang, Dung H. Nguyen, Minh D. Pham, Binh N. Do, Hoang C. Nguyen, Linh V. Pham, Lien T. H. Nguyen, Hoi T. Nguyen, Nga T. Trieu, Thinh V. Do, Manh V. Trinh, Tung H. Ha, Dung T. Phan, Thao T. P. Nguyen, Kien T. Nguyen and Shwu-Huey Yang

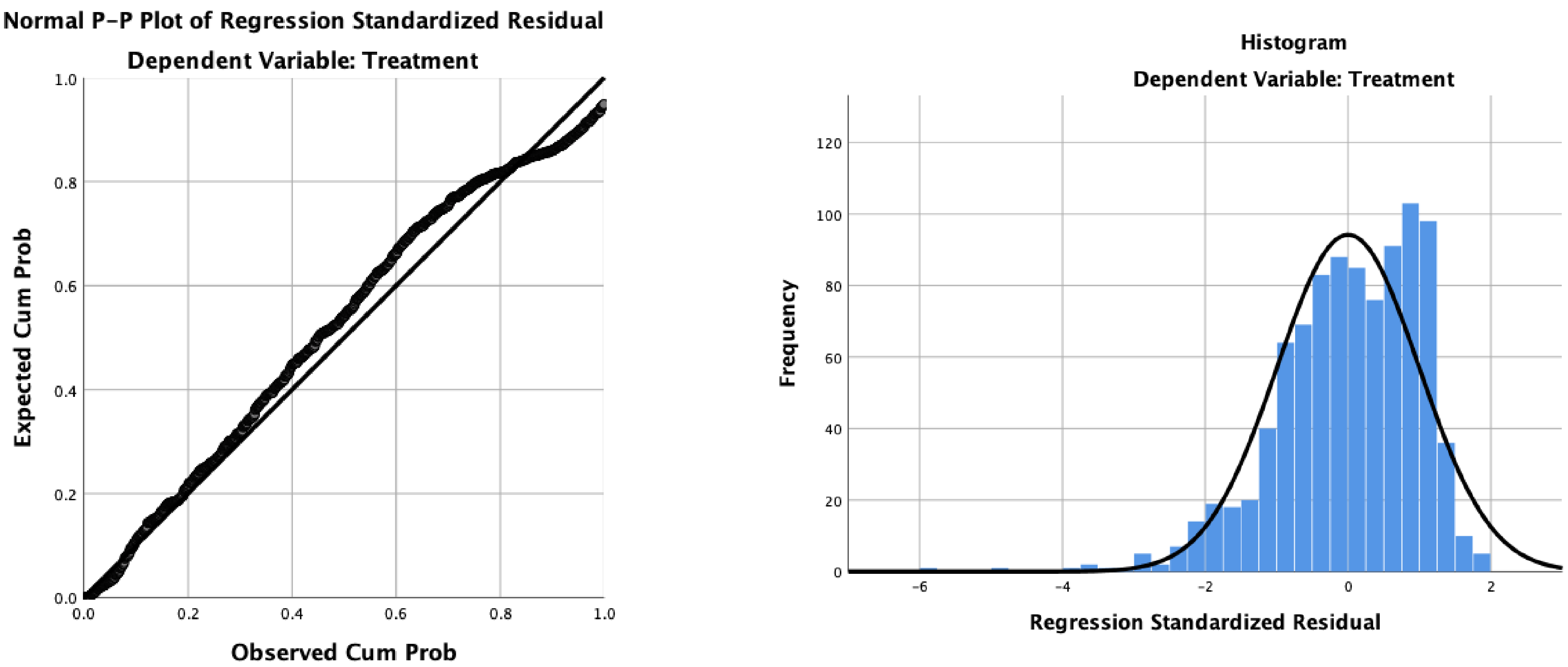

Figure S1. The assumption checked for linear regression models of treatment adherence. Normality: The residuals follow a normal distribution

Table S1. The correlations between the independent variable (Treatment Adherence).

	**Age**	**Education**	**Working Status**	**Social Status**	**Medication Payment Ability**	**S. COVID 19-S**	**HD Vintage**	**CCI**	**HL**	**DDL**	**Fear of Covid-19**
Age	1.000										
Education	-0.093	1.000									
Working Status	-0.030	0.350	1.000								
Social Status	-0.030	0.317	0.212	1.000							
Medication Payment Ability	0.043	0.189	0.148	0.287	1.000						
S. COVID 19-S	0.077	0.038	0.087	-0.024	0.063	1.000					
HD Vintage	-0.013	-0.001	-0.041	0.016	-0.060	-0.114	1.000				
CCI	0.192	0.003	0.030	-0.111	-0.145	0.211	-0.018	1.000			
HL	-0.155	0.236	0.166	0.181	0.212	0.050	-0.037	-0.117	1.000		
DDL	-0.146	0.251	0.140	0.081	0.170	-0.006	-0.018	-0.141	0.747	1.000	
Fear of Covid 19	0.016	-0.027	0.013	-0.139	-0.254	-0.121	0.014	0.181	-0.108	-0.051	1.000

Abbreviation: S-COVID-19-S, suspected COVID-19 symptoms; HD, Hemodialysis; CCI, Charlson Comorbidity Index.; HL, Health literacy; DDL, digital health diet literacy; HDK, hemodialysis dietary knowledge.

Table S2. Associate factors of adherence to hemodialysis treatment, medication, fluid, and diet via bivariate linear regression analysis.

	**Hemodialysis Treatment**		**Medication**		**Fluid and Diet**	
	**B (95% CI)**	**p**	**B (95% CI)**	**p**	**B (95% CI)**	**p**
Age						
19-59						
60-85	2.79 (-7.11, 12.70)	0.580	2.70 (-0.79, 6.27)	0.129	13.23 (0.78, 25.68)	0.037
Gender						
Male						
Female	-7.07 (-16.83, 2.69)	0.155	-0.57 (-4.05, 2.91)	0.747	3.66 (-8.61, 15.94)	0.588
Education						
Illiterate or elementary						
Junior high school	-23.83 (-36.13, -11.52)	<0.001	-1.75 (-5.95, 2.44)	0.412	-3.42 (-18.68, 11.83)	0.660
Senior high school or above	2.07 (-11.05, 15.20)	0.756	5.75 (1.28, 10.22)	0.012	33.71 (17.42, 49.99)	0.001
Working status						
Not working						
Working	0.03 (-10.84, 10.91)	0.995	-1.79 (-5.47, 1.87)	0.337	12.74 (0.01, 25.46)	0.050
Married status						
Never married						
Ever married	-3.21 (-20.37, 13.96)	0.714	-1.88 (-8.01, 4.23)	0.548	11.18 (-10.34, 32.71)	0.308
Social status						
Low						
Middle and high	0.03 (-10.84, 10.91)	0.995	8.07 (4.22, 11.92)	<0.001	26.05 (12.78, 39.32)	0.001
Medication payment ability						
Very or fairly difficult						
Very or fairly easy	11.18 (-0.21, 22.59)	0.055	7.46 (3.41, 11.51)	<0.001	35.81 (21.76, 49.86)	0.001
S-COVID-19-S						
Without S-COVID-19-S						
With S-COVID-19-S	1.37 (-12.21, 14.95)	0.843	- 9.74 (-14.54, -4.94)	<0.001	34.72 (17.76, 51.67)	<0.001
BMI, kg/m^2						
BMI < 24						
BMI ≥ 24	1.52 (-15.13, 18.18)	0.858	-3.05 (-8.97, 2.87)	0.312	12.33 (-8.43, 33.09)	0.244
HD vintage, year						

< 5						
≥ 5	-22.15 (-31.84, -12.46)	<0.001	-8.74 (-12.19, -5.29)	<0.001	-27.24 (-39.45, -15.04)	<0.001
CCI	-3.17 (-6.02, -0.33)	0.029	-0.71 (-1.73, 0.31)	0.172	-1.52 (-5.11, 2.06)	0.406
HL index	0.16 (-0.35, 0.67)	0.537	0.18 (0.00, 0.37)	0.047	1.61 (0.96, 2.24)	0.001
DDL index	0.22 (-0.19, 0.63)	0.301	0.22 (0.07, 0.37)	0.003	1.12 (0.61, 1.64)	0.001
HDK	1.89 (-0.21, 4.00)	0.078	0.89 (0.14, 1.64)	0.02	-2.71 (-5.37, -0.06)	0.045
Fear of COVID-19	-0.26 (-1.07, 0.54)	0.517	-0.21 (-0.49, 0.08)	0.159	-2.57 (-3.57, -1.56)	0.001

Abbreviation: B, regression coefficient; CI, confidence interval; BMI, body mass index; S-COVID-19-S, suspected Covid-19 symptoms; HD, Hemodialysis; HL, health Literacy; DDL, digital health diet literacy; HDK, hemodialysis dietary knowledge; CCI, Charlson Comorbidity Index.

Table S3. The correlations between the independent variable (Hemodialysis treatment).

	**Gender**	**Education**	**Medication Payment Ability**	**HD Vintage**	**HDK**	**CCI**
Gender	1.000					
Education level	-0.085	1.000				
Medication Payment Ability	-0.047	0.189	1.000			
HD Vintage	0.042	-0.001	-0.060	1.000		
HDK	-0.011	-0.085	0.155	0.087	1.000	
CCI	0.019	0.003	-0.145	-0.018	-0.131	1.000

Abbreviation: HD, Hemodialysis; CCI, Charlson Comorbidity Index; HDK, hemodialysis dietary knowledge.

Table S4. The correlations between the independent variable (Medication treatment).

	Age	Education	Social Status	Medication Payment Ability	S. COVID19	HD Vintage	CCI	HL	DDL	Fear COVID	HDK
Age	1.000										
Education level	-0.093	1.000									
Social Status	-0.030	0.317	1.000								
Medication Payment Ability	0.043	0.189	0.287	1.000							
S. COVID 19	0.077	0.038	-0.024	0.063	1.000						
HD Vintage	-0.013	-0.001	0.016	-0.060	-0.114	1.000					
CCI	0.192	0.003	-0.111	-0.145	0.211	-0.018	1.000				
HL	-0.155	0.236	0.181	0.212	0.050	-0.037	-0.117	1.000			
DDL	-0.146	0.251	0.081	0.170	-0.006	-0.018	-0.141	0.747	1.000		
Fear Covid	0.016	-0.027	-0.139	-0.254	-0.121	0.014	0.181	-0.108	-0.051	1.000	
HDK	0.050	-0.085	0.037	0.155	-0.036	0.087	-0.131	0.165	0.181	-0.020	1.000

Abbreviation: S-COVID-19-S, suspected COVID-19 symptoms; HD, Hemodialysis; CCI, Charlson Comorbidity Index; HL, Health literacy; DDL, digital health diet literacy; HDK, hemodialysis dietary knowledge.

Table S5. The correlations between the independent variable (Fluid and Diet).

	Age	Education	Social Status	Medication Payment Ability	S. COVID19	HD Vintage	HL	DDL	Fear COVID	Working status	HDK
Age	1.000										
Education level	-0.093	1.000									
Social Status	-0.030	0.317	1.000								
Medication Payment Ability	0.043	0.189	0.287	1.000							
S. COVID 19	0.077	0.038	-0.024	0.063	1.000						
HD Vintage	-0.013	-0.001	0.016	-0.060	-0.114	1.000					
HL	-0.155	0.236	0.181	0.212	0.050	-0.037	1.000				
DDL	-0.146	0.251	0.081	0.170	-0.006	-0.018	0.747	1.000			
Fear Covid	0.016	-0.027	-0.139	-0.254	-0.121	0.014	-0.108	-0.051	1.000		
Working status	-0.03	0.35	0.212	0.148	0.087	-0.041	0.166	0.14	0.013	1.000	
HDK	0.05	-0.085	0.037	0.155	-0.036	0.087	0.165	0.181	-0.02	0.021	1.000

Abbreviation: S-COVID-19-S, suspected COVID-19 symptoms; HD, Hemodialysis; HL, Health literacy; DDL, digital health diet literacy; HDK, hemodialysis dietary knowledge.
